# Nutritional interventions in children and adolescents with cerebral palsy: systematic review

**DOI:** 10.1590/1984-0462/2024/42/2022107

**Published:** 2023-07-10

**Authors:** Danielle Cristina Guimarães da Silva, Marcela de Sá Barreto da Cunha, Amanda de Oliveira Santana, Augusto Matheus dos Santos Alves, Marcos Pereira Santos

**Affiliations:** a Universidade Federal do Oeste da Bahia, Barreiras, BA, Brasil.

**Keywords:** Cerebral palsy, Nutritional therapy, Malnutrition, Review, Paralisia cerebral, Terapia nutricional, Desnutrição, Revisão

## Abstract

**Objective:**

To systematically review the literature in search of the most suitable and effective nutritional interventions and indications for the nutritional treatment of children and adolescents with cerebral palsy (CP).

**Data source:**

This review was conducted in accordance with the Preferred Reporting Items for Systematic Reviews and Meta-Analyses (PRISMA) guidelines. The articles were selected from seven databases (Cochrane, Literatura Latino-Americana e do Caribe em Ciências da Saúde — Lilacs, Embase, United States National Library of Medicine — PubMed, Scientific Electronic Library Online — SciELO, Scopus, and Web of Science). Studies from a pediatric group (0 to 18 years old) diagnosed with CP were included and the search strategy included the descriptors: “children” OR “childhood” AND “nutritional therapy” OR “nutritional intervention” OR “nutrition” OR “nutritional support” OR “diet” AND “cerebral palsy” OR “cerebral injury”. Methodological quality was assessed using the checklist for cross-sectional analytical studies, the Newcastle-Ottawa scale or the Cochrane Collaboration tool for clinical trials.

**Data synthesis:**

Fifteen studies (n=658) published from 1990 to 2020 met the inclusion criteria. All of them had a low risk of bias. The data showed that children and adolescents with CP have worse nutritional status than those normally developed. Those who received hypercaloric and hyperprotein nutritional supplementation benefited from its use. Studies indicate that enteral nutrition should be considered when nutritional needs are not met by the oral diet, especially in cases where oral motor functions are impaired. In addition, there was a direct relationship between the consistency of food, the level of motor function and nutritional status.

**Conclusions:**

Children and adolescents with CP have a greater risk of malnutrition. The use of nutritional supplementation may help with weight gain. In addition, enteral nutrition and modification of food texture have been used to improve the nutritional status of this group.

## RESUMO

**Objetivo:**

Revisar sistematicamente a literatura em busca das intervenções e indicações nutricionais mais adequadas e eficazes para o tratamento nutricional de crianças e adolescentes com paralisia cerebral (PC).

**Fontes de dados:**

Esta revisão foi conduzida de acordo com as diretrizes Preferred Reporting Items for Systematic Reviews and Meta-Analyses — PRISMA. Os artigos foram selecionados em sete bases de dados (Cochrane, Literatura Latino-Americana e do Caribe em Ciências da Saúde — Lilacs, Embase, United States National Library of Medicine — PubMed, Scientific Electronic Library Online — SciELO, Scopus, and Web of Science). Foram incluídos estudos de um grupo pediátrico (zero a 18 anos) com diagnóstico de PC e a estratégia de busca incluiu os descritores: “children” OR “childhood” AND “nutritional therapy” OR “nutritional intervention” OR “nutrition” OR “nutritional support” OR “diet” AND “cerebral palsy” OR “cerebral injury”. A qualidade metodológica foi avaliada utilizando a lista de verificação para estudos transversais analíticos, escala Newcastle-Ottawa ou ferramenta da Cochrane Collaboration, para ensaios clínicos.

**Síntese dos dados:**

Quinze estudos (n=658) publicados de 1990 a 2020 preencheram os critérios de inclusão. Todos tiveram baixo risco de viés. Os dados mostraram que crianças e adolescentes com PC apresentam pior estado nutricional do que os normalmente desenvolvidos. Aqueles que receberam suplementação nutricional hipercalórica e hiperproteica beneficiaram-se de seu uso. Estudos demonstram que a nutrição enteral deve ser considerada quando as necessidades nutricionais não são supridas pela dieta oral, principalmente nos casos em que as funções oromotoras estão prejudicadas. Além disso, houve relação direta entre a consistência dos alimentos, o nível de função motora e o estado nutricional.

**Conclusões:**

Crianças e adolescentes com PC têm maior risco de desnutrição. O uso de suplementação nutricional pode auxiliar no ganho de peso desses pacientes. Além disso, a nutrição enteral e a modificação da textura dos alimentos têm sido artifícios utilizados para a melhora do estado nutricional neste grupo.

## INTRODUCTION

Nutritional interventions in children and adolescents with cerebral palsy (CP) are an important strategy for maintaining the overall health of this group, with a view to preventing nutritional deficiencies and malnutrition and promoting health.

CP was described for the first time in 1843, after a study was conducted of 47 children with a similar clinical status who suffered from seizures at birth. Currently, CP is defined as a group of postural and movement disorders that limit activities. It occurs due to non-progressive alterations in the development of the fetus’ or infant’s brain.^1,2^ According to World Cerebral Palsy, around 17 million people around the world have CP and the disorder affects two in every thousand live births.^3,4,5^

Generally, the diet of individuals with CP should be similar to that of healthy people, given that their needs involve maintaining adequate nutrition in terms of quantity and quality.^6^ However, in situations of gastroesophageal reflux or difficulties with chewing and swallowing, diet modifications are needed.^7^ It should be noted that the type and severity of CP can directly influence nutritional status. Moreover, abnormal muscle tone can cause greater energy expenditure.^8^

National and international studies have shown that children and adolescents with CP have an increased risk of malnutrition.^7,8,9,10,11^ Thus, it is essential to identify risk factors for this clinical condition early in order to improve the prognosis of these individuals. Epidemiological investigations have presented different behaviors for the nutritional therapy of individuals with CP, ranging from recommending the use of nutritional supplements to the best route for administering the diet. These indications directly influence the choice of nutritional therapy that will be used, as starting the ideal nutritional behavior early is essential to improve the prognosis of the group in question.^12–14^

Despite the importance of this topic, there are a limited number of systematic review studies on the behaviors and influence of nutritional interventions on nutritional outcomes, for example nutritional deficiencies and malnutrition, in cases of CP. Therefore, the main objective of this review is to present the most suitable and effective nutritional interventions and indications for the nutritional treatment of children and adolescents with CP.

## METHOD

This systematic review followed the Preferred Reporting Items for Systematic Reviews and Meta-Analyses (PRISMA) guidelines,^15^ using studies that have assessed nutritional interventions, dietary characteristics, and recommended nutritional therapies for CP in infants. The protocol for this systematic review was registered in the International Prospective Register of Systematic Reviews (PROSPERO) database (CRD 42020175068).

Articles, theses, and dissertations were chosen that used observational studies or clinical trials to assess the efficacy of nutritional interventions or therapies and their effect in children and adolescents (zero to 18 years old) with CP from around the world. Books, book reviews, editorials, review articles, and case reports were excluded.

**Figure 1.**
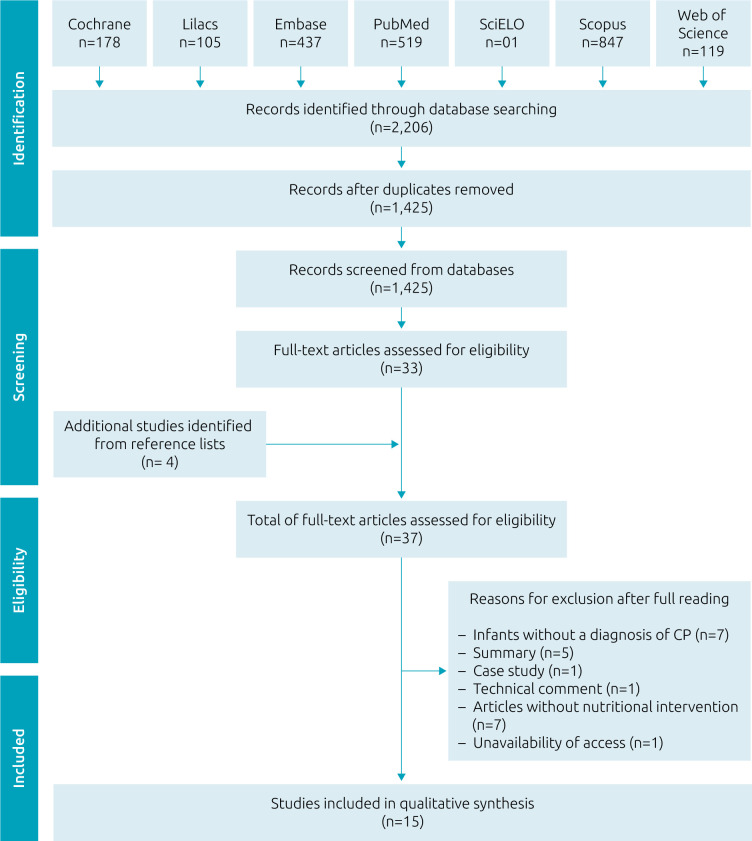
Flow diagram of literature search and selection criteria.

Electronic searches were carried out in the Cochrane, Literatura Latino-Americana e do Caribe em Ciências da Saúde — Lilacs, Embase, United States National Library of Medicine — PubMed, Scientific Electronic Library Online — SciELO, Scopus, and Web of Science electronic databases, and in the lists of bibliographical references of the articles and other reviews on the subject, in order to identify works that were not indexed in the databases, but that would be important to include in this review (Figure 1)^1^. Published articles registered in the databases until October 2020 in Portuguese, English and Spanish, were included. They were identified using the descriptors (“children” OR “childhood”) AND (“nutritional therapy” OR “nutritional intervention” OR “nutrition” OR “nutritional support” OR “diet”) AND (“cerebral palsy” OR “cerebral injury”).

Two independent reviewers identified, selected, and extracted the data from the eligible studies. First, they screened the articles by reading the titles and abstracts. Then they read the eligible ones in full, in order to make the final selection. The reviewers discussed the eligibility and exclusion criteria and defined the final selection by consensus. Some relevant information (authors, year of publication, country of publication, sample size, study type, age and sex of the children, feeding route, nutritional recommendations used, and main results) were systematically organized on an Excel spreadsheet. All the references were managed using the Rayyan application, developed by the Qatar Computing Research Institute (QCRI).^16^ The data were presented via descriptive tables containing the main results of the studies.

The risk of bias was assessed using the following protocols: the checklist for analytical cross-sectional studies,^17^ critical appraisal of cohort studies, critical appraisal of case-control studies, the Newcastle-Ottawa scale,^18^ and the Cochrane Collaboration tool for assessing risks of bias in clinical trials.^19^

## RESULTS

A total of 2,206 articles were identified, of which 1,425 were duplicates. After the initial screening, 748 were excluded by reading the titles and abstracts. The researchers examined the references of the 33 articles chosen to read in full, which led to the identification of four more studies, resulting in 37 inclusions (Figure 1). Of these publications, 15 were eligible for the study^12–14,20–31^ as they contained information about nutritional interventions or indications for nutritional therapies for infants with CP. The main reasons for exclusion were the following: infants with no diagnosis of CP (n=7), abstracts lacking information (n=5), case study (n=1), technical commentary (n=1), articles with no nutritional intervention (n=7), and not available to access (n=1).

The eligible studies were carried out in six countries: Australia,^13,22,28,31^ Brazil,^23,24^ the United States,^27^ Mexico,^12,21,25^ the United Kingdom,^26,29,30^ and Turkey.^14,20^

A predominance of cohort studies was verified, which accounted for seven publications,^14,21,25–27,29,30^ followed by three studies characterized as case-control,^13,20,31^ three cross-sectional studies,^22–24^ and two randomized clinical trials.^12,28^ There was a predominance of publications that addressed the types of spastic, dyskinetic and ataxic CP^13,20–24,27–29,31^ and five studies presented information only on spastic CP.^12,14,25,26,30^

The studies analyzed involved a total of 658 children and adolescents, aged between zero and 18 years old, of both sexes, diagnosed with CP. Studies published between 1990 and 2020 were included, with a predominance of publications between 2008 and 2020 (73%). The population was recruited through the tertiary healthcare network or through the institutions where the subjects were receiving or had received some type of care. Twelve publications were identified in which the participants received their nutritional therapy at home and were monitored in hospital visits.^12,13,20–24,27–30^ In two studies, the participants received their nutritional therapy at home and were monitored in hospital visits or home visits by the team responsible for the study,^14,26^ and only in one did the children and adolescents receive their nutritional therapy and monitoring at a hospital institution.^25^

In relation to the methodological quality of the studies, for the cross-sectional studies it was verified that all three (100%) publications clearly defined the inclusion criteria, they described the subjects and scenarios in detail, they measured exposure in a valid and reliable way, they used objective and standard criteria to measure the condition of the children, and they measured the results in a valid and reliable way.^22–24^ In only one publication (33.3%) was it identified that strategies were used to address confounding factors^22^ and two studies (66.6%)^22,24^ used appropriate statistical analysis to address the confounding factors. No study was excluded due to its methodological quality.

According to the Newcastle-Ottawa scale used to assess methodological quality, the scores varied between 6 and 8, so all the articles had a low risk of bias. The main problems with the methodological quality of the cohort and case-control studies were related to category 2 (comparability between the exposed and non-exposed individuals), as 90% (n=9) of these did not address this.^13,14,20,21,25–27,29–31^

**Figure 2.**
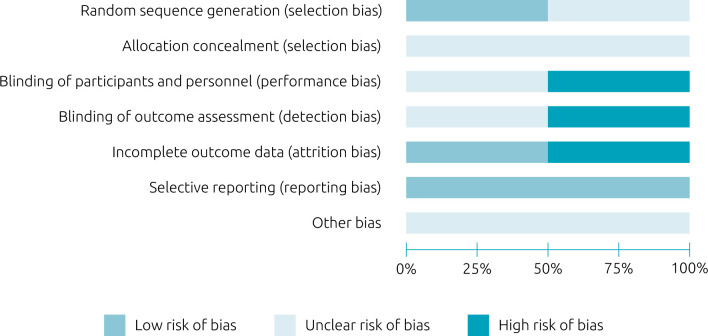
Risk of bias for clinical trials using the *Cochrane risk-of-bias tool for randomized trials* (RoB 2).^32^

**Figure 3.**
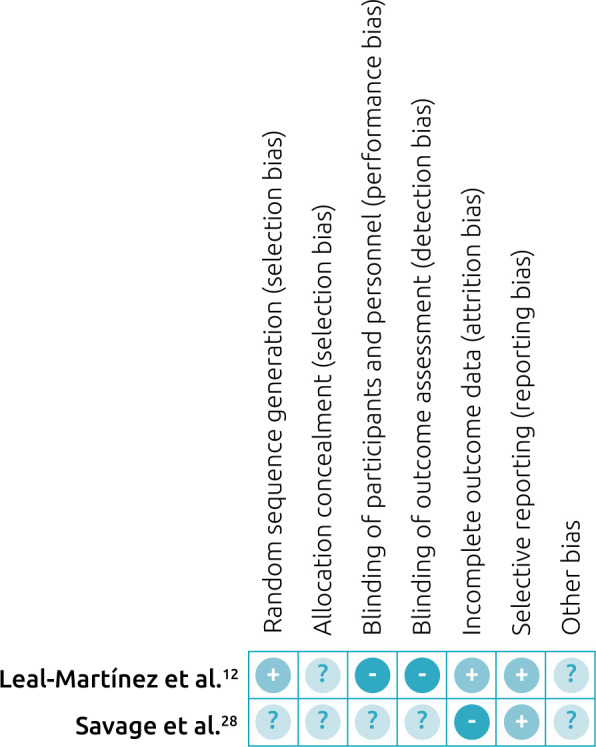
Summary of risk of bias.

The risk of bias assessment of the clinical trials is summarized in Figure 2.^32^ Two clinical trials^12,28^ were assessed using the Cochrane risk-of-bias tool for randomized trials (RoB 2).^32^ In the generation of a random sequence domain, only one of the studies^12^ described the random selection of the sample. For the concealed allocation domain, none of the studies clearly described the form of allocation concealment. For performance bias, although one of the studies^28^ reported being double blind, there were no details about the blinding of participants and of professionals, and it was classified as “uncertain risk of bias.” In the study of Leal-Martínez et al.,^12^ there was no mention of blinding of professionals or of participants, and it was classified as “high risk of bias”. Due to a sampling loss of around 15%, which could influence the statistical analyses of the outcomes, one of the studies was classified as having a high risk of attrition bias.^28^ Finally, all the studies included in this review were free of reports of selective outcomes, because this bias was one of the exclusion criteria. Figure 3 ^12,28^ presents a detailed assessment for each domain of risk of bias in the studies.

This review identified nutritional interventions that used only the oral route in five articles^12,14,20,21,26^ and interventions that used only the enteral route in six publications,^23,25,27–30^ two of which used a nasogastric tube or gastrostomy^25,27^ and four of which used gastronomy. The other four publications used a combination of the oral and enteral routes.^13,22,24,31^ Six eligible studies proposed some type of nutritional supplementation for CP (Table 1).^12–14,20–31^

**Table 1.** Results of studies.

Reference	Interventions (feeding route, nutritional recommendations used)
Arslan et al.,^20^	Oral feeding. Normal diet for the children in the control group. Differentiated consistency diet for the children in the orally fed case group.
Barrón-Garza et al.,^21^	Oral feeding for 3 years and 9 months. Hypercaloric diet, with 50% carrying out oral motor therapy with their parents at home and the other half doing so at a clinic.
Benfer et al.,^22^	Oral feeding (n=87) and enteral feeding (n=12).
Brant et al.,^23^	Enteral feeding via gastrostomy, with a mean monitoring period of 5.9 months.
Caselli et al.,^24^	Enteral feeding via gastrostomy (n=25) and oral feeding (n=29).
García-Contreras et al.,^25^	Enteral feeding via nasogastric tube (n=8) and gastrostomy (n=5) for 4 weeks. Use of a lactose-free infant formula (Nestlé^®^), adding corn syrup to the formula to increase the energy density.
Kibirige^26^	Oral feeding for 8 weeks. 4 children received liquid nutrition and supplementation.
Leal-Martínez et al.,^12^	Oral feeding. Use of 3 liquid diets, 2 administered at breakfast and 1 at dinner. Follow-up group (FG) (n=10): normal diet. Control group (CG) (n=10): vermifugated and received a nutritional therapy recommended by the WHO (2010). Intervention group (IG) (n=10): vermifugated and received the Nutritional Support System (NSS).
Sanders et al.,^27^	Enteral feeding via nasogastric tube (n=4) and gastrostomy (47) for 6 months. The dietary formulas used were Complete Modified Formulas (Sandoz) or lsocal (Mead Johnson), each one providing 1 kcal/mL.
Savage et al.,^28^	Enteral feeding by nasogastric tube (n=1) and percutaneous endoscopic gastrostomy (n=12). Standard enteral formula based on casein and another with 50% whey and 50% total casein protein for 1 week (n=7), and a formula based on partially hydrolyzed whey protein (n=6).
Schoendorfer et al.,^31^	Children with CP (case group): percutaneous (n=9) and oral (12) enteral feeding.
Schoendorfer et al.,^13^	Enteral (n=9) and oral (n=15) feeding.
Soylu et al.,^14^	Oral feeding for 6 months.
Sullivan et al.,^29^	Enteral feeding by gastrostomy for 12 months.
Vernon-Roberts et al.,^30^	Enteral feeding by gastrostomy for 6 months. An enteral feeding of low caloric energy (Kcal), complete with micronutrients and rich in fiber, was administered.

With respect to the motor skills and limitations in the feeding process of the children and adolescents, nine publications^12–14,20,25,28–31^ used the Gross Motor Function Classification System (GMFCS) criterion to analyze these and in four studies all the children’s ability to eat was severely compromised.^13,25,28,31^ Six publications did not mention the use of criteria to classify the motor function of children and adolescents with CP.

One publication employed the Development of Care for Individuals with Serious Deficiencies (Dash-2) method,^21^ and four studies applied no methodologies to calculate motor function, basing their assessments on brain damage and nutritional status diagnoses.^23,24,27,29^

Regarding the intervention based on daily elemental iron supplementation indicated by García-Contreras et al.,^25^ no inherent benefits of its used were demonstrated; however, there was an improvement in the nutritional status and an increase in the weight of the children and adolescents. After supplementing the normal diet of children with CP using a hypercaloric and hyperproteic dietary formula with a high biological value, combined with oral motor physiotherapy, Barrón-Garza et al.^21^ identified an increase in weight, which was significantly higher for those who carried out their physiotherapy at the clinic compared to those who did it at home with their parents.

Kibirige^26^ identified greater weight gain in the infants who, besides a normal diet, received supplementation with a hypercaloric and normoproteic formula. Vernon-Roberts et al.^30^ found a significant increase in body weight and other anthropometric measures after supplementing children with CP with a hypocaloric and nutritionally complete enteral formula.

Brant et al.^23^ found a significant increase in body weight from using a nutritionally complete normocaloric formula, maize flour, and oatmeal in combination with a normal diet or a diet composed of soybean oil, albumin, sugar, and soymilk, together with a mineral and vitamin supplement. Leal-Martínez et al.^12^ identified that the diet based on homemade shakes with functional foods (a large amount of vegetables, fruits, cereals, roots, and fish) was positively reflected in improved motor parameters such as walking and standing up.

Two publications analyzed the textures and consistencies of the foods provided to children with CP. Arslan et al.^20^ note that foods with liquid or pasty textures are unable to meet the caloric and nutritional needs of children with CP. For Benfer et al.,^22^ children with CP are unable to eat many foods due to their textures, but such modifications guarantee a more efficient calorie intake, even if it is densely inferior. Although eight studies did not seek to evaluate the textures and consistencies of the foods provided using specific methods,^12,13,23,27–31^ general motor functions served as parameters to define them. In those cases, enteral feeding was often related to severe cases of CP in children and adolescents, and even for those who ate orally, their care was intensified in order to avoid dysphagia and choking.

## DISCUSSION

The results of this study indicate that children and adolescents with CP have a more compromised nutritional status that those who do not have the disorder. Nutritional supplementation of a normal diet leads to weight gain and improved nutritional status in this group. We observed that enteral feeding was used as the main behavior for nutritional treatment. Moreover, the modification of the texture of foods, transforming them into purees and/or semi-solids, was essential for improving the nutritional status of this infant group and for better adapting them to oral consumption.

In a case-control study carried out in Turkey using 85 children with CP, Arslan et al.^20^ identified a greater prevalence of malnutrition and a lower height in children with CP than in the control group. The case-control study of Arrowsmith et al.^33^ carried out in Australia using 167 children corroborates the results of Arslan et al.,^20^ given that the outcome of their study showed that children with CP had a lower body weight, height, and body fat percentage than children without CP. It is suggested that these results are due to the difficulties with chewing and swallowing and the gastrointestinal problems that this population often has.^7^

Another point to mention is that the different feeding routes can directly influence the nutritional therapy of children with CP.^34^ Sullivan et al.^29^ reported that patients who use gastrostomy (GTT) had a greater daily dietary intake, achieving 69% of the recommended energy value, while those fed orally achieved 57% of the recommended energy intake, with less than 10% difference being identified between the two groups. In line with the previous findings, Caselli et al.^24^ analyzed 54 patients with CP fed via different routes — 25 fed via GTT and 29 orally — and concluded that there was no significant difference regarding the prevalence of malnutrition in relation to weight or body mass index. Thus, it is suggested that children with GTT experience less energy expenditure as a result of the lower demand for energy than those fed orally.^35,36^

Regarding the use of supplementation, we observed an improvement in the nutritional status of the children that used nutritional supplementation, especially when the weight gain factor is analyzed.^23,25,27,28^ From this perspective, Vernon-Roberts et al.^30^ carried out a cohort study in the United Kingdom of 14 children with CP and identified body weight gain and linear growth as a result of supplementation with a hypercaloric enteral formula (0.75 kcal/mL) that was nutritionally complete and rich in fibers. The randomized clinical trial carried out in Mexico by Leal-Martínez et al.^12^ found that nutritional supplementation in the form of homemade shakes with functional foods (a large amount of vegetables, fruits, cereals, roots and fish) significantly improved the motor function of children with CP. In a study on the nutritional management of children with CP, Bell et al.^35^ note that first-line treatment involves the inclusion of oral nutritional support adapted to the individual needs of each child, provided they can consume a diet orally without the risk of aspiration or greater complications.

The administration of nutritional support should be adapted to the physical status of the children, their capacity to consume foods, and the possibility of oral ingestion, as their diet needs to be adapted in order to maximize dietary efficiency and reduce fatigue during meals, thus leading to a greater dietary intake.^10,35^ For this, the use of enteral nutrition should be considered when oral feeding does not satisfy their needs and in the case of severe malnutrition and deglutination disorder. This can also be used to complement oral nutrition.^29,35,37^ It is important to stress that nutritional supplementation can improve the prognosis of the group studied and provide a better quality of life.

It is worth highlighting that in the absence of specific nutritional recommendations for children with CP, it is appropriate to use guidelines and nutritional evidence for children with neurological disorders. It also warrants mentioning that malnutrition and nutritional deficiencies in the population studied can be influenced by nutritional and non-nutritional factors.^38^

In their guide “Recommendations for Nutritional Management of Children with Neurological Impairment (NI),” the European Society for Paediatric Gastroenterology, Hepatology, and Nutrition (ESPGHAN) discusses recommendations regarding the nutritional needs of children with intellectual impairment and provides alternative recommendations for children with CP, which can be used when there are no specific parameters for this population.^39^ However, as an alternative for supplementation, foods rich in nutrients that are lacking in the patient can be added to the diet. Nutritional deficiency should be diagnosed via laboratory tests and it is only after identifying a particular deficiency that some type of supplementation should be prescribed.^35,40^

Choosing the right nutritional formula is extremely important for the effectiveness of the nutritional treatment of children with CP. A variety of products is used for nutrition, including polymeric, semi-elemental, and elemental formulas adapted to every need and different life phases. Most enteral foods are formulated to serve as a source of complete nutrition, providing all the nutrients needed for the day-to-day maintenance of the body.^41^ Vernon-Roberts et al.^30^ note that nutritional treatment should be based on the provision of a diet with a standard energy density calculated based on the patient’s age. This should evolve to a high-density formula when the child does not tolerate large quantities of a food. Finally, a lower energy density formula can be used in those who present more rapid weight gain. Thus, it is stressed that the nutritional behavior should be individualized and prescribed according to the particularities of each child.

Some nutritional interventions for CP can be carried out by modifying the textures/consistencies of foods. Such alterations may be recommended to provide safety and efficiency during meals or to stimulate the development of motor oral skills in this population.^42^ In a study of 99 children with CP, Benfer et al.^22^ identified that modifying the texture of foods/fluids in 39% of the population studied led to an improvement in energy and nutritional intake and more autonomy among the children. The authors also showed that the children with modified diets had greater gross motor limitations and were therefore unable to feed themselves with foods in their normal textures. Similarly, Arslan et al.^20^ identified that children with CP who consumed liquid, pasty, or minced diets achieved greater growth and nutritional status, as well as having fewer dietary problems. Thus, exploring the textures of the foods consumed by children with CP will support better management decisions regarding the nutritional therapy and recovery of these patients.

This review study has some limitations. The summary of the results of studies using different methodological approaches was complex and impeded a meta-analysis from being carried out. On the other hand, the age range (0–18 years old) makes it hard to compare and summarize the results. These limitations notwithstanding, we adopted consistent methodological procedures performed by independent reviewers and assessed the studies that fulfilled the eligibility criteria in order to reduce the possibility of bias.

It is concluded that children and adolescents with CP have a greater risk of malnutrition and that the use of nutritional supplementation may help with weight gain. In addition, enteral nutrition and modification of food texture have been used to improve the nutritional status of this group. The nutritional treatment of CP should be multi-factorial and aim to promote a better quality of life and improvement in the prognosis of these individuals, given that diet is an essential factor for the recovery and repair of these patients. To guarantee the success of interventions, nutritional status should be periodically monitored by a multidisciplinary team. For this, we recommend conducting more studies on nutritional interventions in children and adolescents with CP, given that more information is needed to achieve precise and adequate nutritional behavior for this target population.

## Data Availability

The database that originated the article is available with the corresponding author.
